# Validation of the Italian Translation and Cultural Adaptation of the Canadian Assessment of Physical Literacy-2 (CAPL-2) Questionnaire for Children

**DOI:** 10.3390/children12101290

**Published:** 2025-09-24

**Authors:** Alice Iannaccone, Alessandro Cudicio, Lavinia Falese, Bruno Federico, Matteo Crotti, Nicola Lovecchio, Simone Digennaro, Valeria Agosti

**Affiliations:** 1 Department of Human Sciences, Society and Health, University of Cassino and Southern Lazio, Via Sant’Angelo, Loc. Folcara, 03043 Cassino, Italy; l.falese@unicas.it (L.F.); b.federico@unicas.it (B.F.); s.digennaro@unicas.it (S.D.); 2 European University of Technology EUt+, European Union; 3 Department of Human and Social Sciences, University of Bergamo, 24129 Bergamo, Italy; alessandro.cudicio@unibg.it (A.C.); matteo.crotti@unibg.it (M.C.); nicola.lovecchio@unibg.it (N.L.); 4 Department of Humanities, Philosophy and Education, University of Salerno, 84084 Fisciano, Italy; vaagosti@unisa.it

**Keywords:** assessment, cross-cultural adaptation, children, physical literacy, physical education, physical activity, sport, active lifestyle, lifelong, health

## Abstract

**Highlights:**

**What are the main findings?**

- The Italian version of the CAPL-2 questionnaire demonstrated excellent internal consistency in the Motivation and Confidence domain (Cronbach’s α: 0.88–0.97), aligning with international validation studies.
- The questionnaire showed high predictive validity for identifying children who meet WHO recommendations for physical activity, with AUC values of 0.95 (5 days) and 0.89 (6 days).

**What is the implication of the main finding?**

- The validated CAPL-2 offers Italian educators and researchers a culturally adapted, reliable tool to assess children’s physical literacy in school and sport contexts.
- This tool can support early interventions promoting physical activity and lifelong health, in line with Italy’s recent educational reforms and international public health goals.

**Abstract:**

Background/Objectives: Physical literacy is a holistic concept promoting lifelong health by considering an individual’s lived experience within their cultural context. This necessitates context-specific conceptualizations and pedagogies, highlighting the need for valid assessment tools for physical and sport educators. The Canadian Assessment of Physical Literacy (CAPL-2) is a well-known validated tool. This study aimed to validate the Italian translation and cultural adaptation of the CAPL-2 questionnaire for children aged 8–12. Methods: The CAPL-2 questionnaire was translated using a forward–backward procedure by bilingual experts. Subsequently, 111 Italian children (57 females, mean BMI 17.9 kg/m^2^) completed the adapted CAPL-2 questionnaire twice over 10 days under supervision. The internal consistency of CAPL-2 was assessed with Cronbach’s alpha. ROC curve analysis and AUC evaluated the CAPL-2’s ability to predict adherence to WHO physical activity guidelines based on self-reported activity. Results: Results showed high internal consistency for the motivation and confidence domain (Cronbach’s α: 0.88–0.97) but lower consistency for the knowledge and understanding domain (Cronbach’s α: 0.20–0.34). Despite this, the CAPL-2 questionnaire demonstrated high predictive performance in identifying children active for at least 5 days (AUC: 0.95) or 6 days (AUC: 0.89). Conclusions: The Italian version of CAPL-2 is a reliable tool for assessing physical literacy in Italian children aged 8 to 12, addressing key aspects such as motivation, confidence, physical skills, understanding of physical activity, and daily habits. It offers a valuable and culturally adapted instrument for trainers, teachers and educators in physical activity and sport contexts.

## 1. Introduction

The youngest generations are increasingly dedicating their time to sedentary activities, such as browsing social media, which has negative consequences for their overall well-being [1,2,3]. This is particularly troubling in relation to their development of identity, body image, and emotional intelligence. The rise in sedentary behaviour results in lower levels of physical activity, potentially worsening the adverse effects of unhealthy lifestyles [4,5,6]. Conversely, regular physical activity (PA) is associated with substantial physical and mental health benefits [7,8,9]. Among children and adolescents, physical activity (PA) supports healthy musculoskeletal development, particularly bone health, motor skill acquisition, and cognitive development. Despite these benefits, physical inactivity remains a global epidemic: 81% of boys and girls aged 11–17 years spend less than one hour a day performing moderate- to vigorous-intensity physical activity (MVPA) [7,9]. To address this public health challenge, global targets set by the WHO aim to reduce physical inactivity by 10% in 2025 and 15% in 2030 compared to 2010 levels, highlighting the urgency for multisectoral strategies due to projected healthcare costs potentially reaching USD 27 billion annually [10,11]. The findings from the recent national Health Behaviour in School-Aged Children (HBSC) survey in Italy reveal critical insights into the physical activity levels of adolescents. Specifically, the survey indicates that a mere 10.8% of male adolescents and 5.4% of female adolescents are meeting the recommended guidelines for MVPA [2].

In the context of promoting physical activity and addressing high rates of physical inactivity, the concept of physical literacy (PL) is globally emerging as a valuable theoretical construct around which to structure effective interventions [10,11]. There is increasing evidence that holistic approaches, such as multicomponent interventions targeting different factors (motivation, knowledge, competence, and self-perception), lead to greater improvements in physical activity than single-factor interventions [12,13].

For instance, Farias et al. [14] conducted a retrospective analysis to investigate the enduring impacts of a Sport Education curriculum on students’ physical literacy (PL) reporting notable changes in students’ motivation, perspectives, and behaviours concerning physical education (PE) and sports. The results revealed that students cultivated empathetic attitudes, developed resilience against discriminatory behaviour, and demonstrated a commitment to fair participation. These skills transcended the school setting, fostering the development of lifelong PL.

PL was described as an inclusive and holistic concept, conceived from its inception to promote lifelong health and well-being by centering on the person-in-the-world and their lived experience within their cultural context [15,16]. More recently, PL was emphasized as a lifelong journey characterized by evolving interactions between motivation, competence, knowledge, and the social and cultural context, highlighting the necessity of culturally appropriate assessment tools to foster physical activity effectively throughout life [15,16].

This concept is now increasingly recognized as a multidimensional construct, encompassing four domains: physical competence, motivation, confidence, knowledge, and behaviors necessary for lifelong engagement in physical activity [17,18], enabling people to participate meaningfully in physical and sport activities across their lifespan, thus aligning with strategies to combat sedentary lifestyles and foster lifelong health [19,20]. By fostering PL from an early age, individuals can develop a foundation that supports sustained physical and sport activity and well-being in adulthood and older age [21]. From these perspectives, PL is not confined to childhood but evolves continuously, adapting to the changing abilities and contexts throughout life [22,23]. Developing robust PL skills early in life helps individuals establish personal strategies to sustain active lifestyles throughout their lifespan [24].

Recognizing that diverse cultures, governance structures, geographical locations, and physical environments necessitate tailored conceptualizations and pedagogies to effectively foster PL [25,26], a recent Global Physical Literacy (GloPL) Action Framework [27] was established to unify this complex dimension in objectives and principles, as well as to delineate effective strategies for advancing PL on a global scale [28].

Standardized assessments of PL that take into account its complex dimensions are crucial to evaluate PL levels in the population and to support PL development [22,24]. The Canadian Assessment of Physical Literacy—Second Edition (CAPL-2) [29] has emerged as a comprehensive tool effective in evaluating multiple aspects of PL and a key instrument for understanding how PL develops and evolves across childhood [22,29,30] within different international contexts [31]. The CAPL-2 assessment comprises three Physical Competence protocols, two Daily Behavior protocols, and a 22-item questionnaire assessing the domains of Motivation and Confidence, and Knowledge and Understanding. Regarding the 22-item questionnaire, crucial for evaluating the cognitive and affective aspects of PL, extensive research has highlighted the necessity of tailoring the CAPL-2 questionnaire to reflect the linguistic nuances and cultural contexts of target populations, ensuring the assessment’s validity and reliability into various languages and contexts [17,20,30,32,33,34,35].

Recognizing the unique cultural and educational characteristics of Italy, where recent investments have been made to enhance primary school physical education by introducing specialized physical education teachers in grades four and five (Legge 234/2021) [36], reflecting increased awareness of the inactivity pandemic [33], this study aims to validate and culturally adapt the CAPL-2 for Italian children, ensuring the instrument’s appropriateness and reliability within the Italian context.

## 2. Materials and Methods

### 2.1. Sample and Procedures

A total of 111 children (54 males; 57 females) aged 8 to 12 years participating in a national summer camp in Italy were recruited (Table 1).

The convenience sample was recruited during a national summer camp attended by children from multiple Italian regions (including Northern, Central, Southern areas, and an island), ensuring heterogeneity in socio-cultural and socio-economic backgrounds and thus providing a broad representation of the national territory. The summer camp context was intentionally chosen because it allowed for continuous attendance of participants, close supervision by trained research assistants, and standardized administration procedures. In addition, this setting provided the optimal opportunity to administer the questionnaire twice within the required 10-day interval for the test-retest reliability assessment, under consistent and controlled conditions.

Eligibility criteria were the ability to speak Italian and an age between 8 and 12 years, consistent with the target population of the CAPL-2 and with previous validation studies of physical literacy questionnaires [29,37]. Importantly, children were not selected on the basis of competitive sport participation or prior knowledge/skills, making the sample representative of the general population of Italian children within this age range.

All research assistants underwent standardized training sessions before data collection, ensuring consistency in questionnaire administration, data collection procedures, and interaction with children. This study was conducted in accordance with the Declaration of Helsinki and approved by the Ethics Committee of the University of Cassino and Southern Lazio—IRB Department of Human Sciences, Society and Health (Approval number: 4R; Approval date: 11 September 2024).

To assess the internal consistency and reliability of the Italian version, the questionnaire was administered to the same group of children on two separate occasions, with a ten-day interval between the first and second administrations. To enhance the confidentiality of assessment results and to ensure the integrity of the test–retest process, each child was assigned a unique identification number, which was used to accurately record the scores obtained. Participants completed the questionnaire under the supervision of trained research assistants, who provided detailed instructions and responded to any inquiries that arose during the process. In accordance with the CAPL-2 guidelines, responses were collected anonymously on paper and subsequently entered into a secure database for analysis by a designated researcher.

### 2.2. CAPL-2 Measures

The current questionnaire is part of the comprehensive protocol developed by the Canadian Assessment of Physical Literacy-2 (CAPL-2) [29,37], designed to accurately and reliably assess a wide array of skills and abilities that define a child’s level of physical literacy.

Specifically, the CAPL-2 questionnaires consist of two key domains of PL (i.e., motivation and confidence; knowledge and understanding domain) and four subscales.

Importantly, according to the CAPL-2 manual, self-reported MVPA is not a separate domain but is included within the broader daily behaviour domain of the CAPL-2 protocol, which encompasses both objective (e.g., pedometer-based) and subjective (self-report) indicators of physical activity.

The first domain, motivation and confidence, assesses children’s confidence in their ability to be physically active and their motivation to participate in physical activity. The total score for this domain is derived from four subscales: Predilection and Adequacy items (“What’s most like me?”), Intrinsic Motivation (“Why are you active?”), and Physical Activity Competence (“How do you feel about being active?”), each consisting of three items. According to the manual, the subscale scores range from 1.5 (or 1.8) to 7.5, with the maximum possible score for the entire domain being 30 points.

The second domain, knowledge and understanding, assesses a child’s knowledge of physical activity, skills and fitness. The assessment comprises four multiple-choice questions, each presenting one correct answer alongside three incorrect options. The second part features a gap-fill task that requires completion of sentences. Each correctly inserted word is worth one point, allowing for a maximum of six points in this section. Overall, the total score for this assessment ranges from 0 to 10, with 10 representing the highest possible score.

In addition, the CAPL-2 questionnaire includes a self-report measure of children’s engagement in at least 60 min of daily MVPA. This item prompts participants to indicate how often they usually achieve this daily activity target. While this self-reported measure represents only one component of the daily behaviour domain, its inclusion provides valuable complementary information and allows for the examination of associations between physical activity levels and other domains of physical literacy.

### 2.3. Translation and Cultural Adaptation

The translation of the CAPL-2 questionnaire into Italian was carried out in accordance with the methodology outlined by Walde & Vollm [38]. This approach ensures accuracy and consistency in the translation process, adhering to the highest standards of linguistic quality.

(1) Translation: The original document was translated into Italian by two researchers who are native speakers, each working independently to ensure accuracy and fidelity to the source material.
(2) Review: The two versions have been systematically compared and reviewed by a researcher with expertise in physical literacy.
(3) Adjudication: Following the feedback provided by the reviewer, a preliminary version of the Italian CAPL-2 questionnaire was finalized.
(4) Pretest: The pre-final version of the Italian questionnaire was administered to a sample of 10 participants to assess the clarity and comprehensibility of the questions. Participants were prompted to provide feedback regarding their understanding of the questionnaire items.
(5) Documentation: After carefully considering the feedback gathered during the pretest phase, the final version of the questionnaire has been officially approved.

A graphical representation of the translation and cultural adaptation process is shown in the Supplementary Material (Figure S1).

The final Italian version of the CAPL-2 questionnaire used for the research is attached as Supplementary Material S1.

### 2.4. Statistical Analysis

All collected data were systematically organized and stored into a database specifically developed for the purposes of this study. The threshold of α = 0.05 was adopted as the level of statistical significance. All calculations were performed using STATA software (v 18, STATACorp, College Station, TX, USA).

The socio-demographic characteristics—such as age, sex, height, body weight, and body mass index (BMI)—and all variables utilized in the calculations were comprehensively detailed. The data were presented as means and standard deviations (SD) for both the test and retest. The normality of all examined variables was evaluated using the Kolmogorov-Smirnov test.

The internal consistency and reliability of the overall domain score, as well as the total scores for each subdomain and individual item outcomes, were assessed using Cronbach’s alpha [39,40]. Furthermore, Spearman’s correlation was employed to examine the relationship between each item and the overall scores for every domain and subdomain.

The assessment of test–retest reliability, or reproducibility, was conducted by calculating the intraclass correlation coefficient (ICC) along with a 95% confidence interval (95% CI). A two-way random effects model was utilized to analyze single measures and absolute agreement, demonstrating the concordance between the initial test and the retest. The interpretation of ICC values adhered to the benchmarks set forth by Landis and Koch [41]: <0.20, slight agreement; 0.21 to 0.40, fair; 0.41 to 0.60, moderate; 0.61 to 0.80, substantial; and >0.80, almost perfect. Additionally, we computed the standard errors of measurement (SEM) and the minimum detectable change (MDC) to assess the error range for each item, subdomain, and domain. Moreover, to check the overall diagnostic performance of the Italian version of CAPL-2 Questionnaire we created a receiver operating characteristic (ROC) curve and evaluated the area under the curve (AUC).

In line with the statistical analysis procedures reported by Knisel et al. [33], two cut-points were selected within the CAPL-2 Questionnaire Manual (the ones that were closer to the World Health Organization PA recommendations for children [9]. The first cut-point concerned engaging in physical activity for at least 60 min for 5 days a week, while the second cut-point concerned engaging in physical activity for at least 60 min for 6 or 7 days a week. A ROC curve analysis was used to assess whether physical literacy scores are predictive of meeting the physical activity cut-points.

## 3. Results

Results showed good to excellent internal consistency for the motivation and confidence domain (Cronbach’s α: 0.88–0.97; mean: 5.3 ± 1.4) and low internal consistency for the knowledge and understanding domain (Cronbach’s α: 0.20–0.34; mean: 9.3 ± 0.98).

Furthermore, the results revealed that the CAPL-2 questionnaire exhibited highly predictive performance in identifying children active for at least 5 days (AUC: 0.95) compared to those active for at least 6 days (AUC: 0.89).

### 3.1. Psychometric Properties of the Questionnaire

Table 2 and Table 3 provide an overview of the internal consistency, reliability, and systematic variations associated with the self-reported physical activity questions, motivation and confidence questions, and the knowledge and understanding questions from the CAPL-2 battery assessment.

The Knowledge and Understanding domain exhibited low internal consistency (Cronbach’s α: 0.20–0.34), likely due to limited variability and the relatively high baseline knowledge demonstrated by the sample, primarily composed of physically active children.

Correlation analysis of Pearson’s r was performed for the first measurement of the tool (test). The results of these analyses are presented in Table 4.

Results showed that the analysis of the subscales of the Motivation and Confidence domain is nearly perfect, and associations were observed regarding the overall score of the Motivation and Confidence domain.

### 3.2. Predictiveness of the CAPL-2 Questionnaire Based on Physical Activity

As demonstrated from previous research [33], the questionnaire allows to check the overall diagnostic performance of the CAPL-2 questionnaire. For this purpose, the ROC curve analysis was performed, and the AUC was estimated. The analysis was made by setting two cut-points [9]: physically active for at least 60 min for 5 days a week; physically active for 6 days a week. The AUC values of the models were sufficient [42], indicating a good fit of the classification (Table 5). The highest predictive capability was found for the model based on the first cut-point.

**Figure 1:**
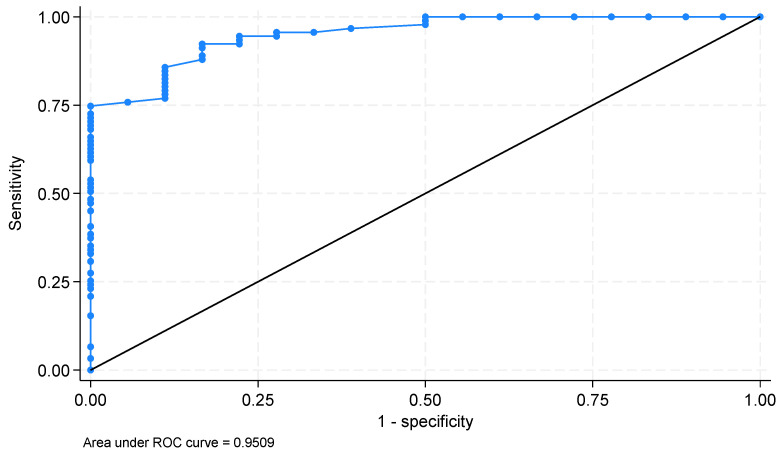
ROC curve for the physical activity cut-off point for 5 days and 60 min.

The ROC curve for the first physical activity cut-point (PA for 5 days and 60 min) is reported in Figure 1.

These results indicate that physical literacy scores are predictive of physical activity levels (engaging in at least 60 min of PA over at least 5 days).

## 4. Discussion

The present study aimed to validate and culturally adapt the CAPL-2 questionnaire for Italian children aged 8 to 12 years. Our findings demonstrate that the Italian version of the CAPL-2 is a reliable and valid instrument for assessing PL in this population.

The Italian adaptation of the CAPL-2 questionnaire demonstrated excellent internal consistency within the Motivation and Confidence domain, with Cronbach’s alpha values ranging from 0.88 to 0.97 across subscales (see Table 2). These findings align with previous validation studies in other cultural contexts. For instance, Elsborg et al. [32] reported high internal consistency in the Danish version of the CAPL-2, supporting the reliability of this domain across different populations. Similarly, Gunnell et al. [43] refined the Motivation and Confidence domain in the CAPL-2, resulting in a shorter, theoretically aligned questionnaire with strong psychometric properties. These consistent findings across studies suggest that the Motivation and Confidence domain is robust and effectively captures the intended constructs in diverse cultural settings [44]. The high item-total correlations underline the questionnaire’s reliability and suggest that the translated and culturally adapted items effectively capture Italian children’s motivational and self-perception dimensions associated with PA.

With respect to the Knowledge and Understanding domain, a low internal consistency was found, with Cronbach’s alpha values ranging between 0.20 and 0.34 (see Table 3), hypothetically explained by the high homogeneity among answers, providing low variability. Nevertheless, results are consistent with previous international validation studies of the CAPL-2 questionnaire. For example, Pastor-Cisneros et al. [17] reported similar results in the Spanish version, suggesting potential challenges in measuring knowledge and understanding consistently across different populations. Similarly, in the Chinese version of CAPL-2, Li et al. [30] reported a Cronbach’s α of 0.52 for the Knowledge and Understanding domain, whereas the Motivation and Confidence domain achieved a significantly higher α of 0.82, indicating that the cognitive domain consistently demonstrates lower internal consistency across cultural adaptations.

A Confirmatory Factor Analysis (CFA) was not performed in the present study due to the relatively small sample size, which would not meet the recommended criteria for stable estimation in a multi-item, multi-factor model [45,46]. Our primary aim was to translate, culturally adapt, and assess the internal consistency and test-retest reliability of the CAPL-2 questionnaire, in line with the methodological approach adopted by other cultural adaptation studies [17,32,33]. The consistently low Cronbach’s α values reported internationally for the Knowledge and Understanding domain suggest that this is a recurring characteristic of the domain rather than a result of factorial misfit.

In our sample, the item “Physical activity guidelines” showed modest correlations (0.61 at test, significantly reduced to 0.10 at retest), while the “Improve sport skills” item also displayed low reliability (0.70 at test and 0.23 at retest). Moreover, the low average inter-item correlations (ranging from 0.11 to 0.34) suggest limited coherence among items. Given the consistently high mean scores (9.3 ± 0.98), the low variability among responses likely reduced internal consistency, suggesting a ceiling effect [47]. However, these findings do not necessarily indicate translation flaws or a lack of comprehension among Italian children. Rather, they may reflect both the characteristics of a well-informed and physically active sample, and the structural limitations of this domain across multiple contexts. In particular, the mixed item formats (multiple choice and gap-filling) and the limited number of items may not sufficiently capture the complexity of children’s knowledge.

The inter-subscale correlations observed in Table 4 showed strong positive relationships among the four subscales of the Motivation and Confidence domain: Adequacy, Predilection, Intrinsic Motivation, and Perceived Physical Competence. These findings are consistent with previous studies that have validated the CAPL-2 in different cultural contexts. For instance, Gunnell et al. [43] conducted a factor analysis to refine the Motivation and Confidence domain, resulting in a model that included these four subscales, each demonstrating strong internal consistency and theoretical alignment with self-determination theory. Their study supports the structural coherence of these constructs within the CAPL-2 framework. Similarly, Elsborg et al. [32] reported high internal consistency within the Motivation and Confidence domain, further corroborating the robustness of these interrelated constructs across different populations. These consistent findings across studies reinforce the validity of the Motivation and Confidence domain in assessing PL among children.

Finally, the CAPL-2 demonstrated excellent predictive validity in identifying children meeting the World Health Organization’s physical activity recommendations. The area under the curve (AUC) values were 0.95 for children active at least five days per week and 0.89 for those active six days per week, both statistically significant (*p* < 0.001). These findings are consistent with previous studies validating the CAPL-2 in various populations. For instance, Mendoza-Muñoz et al. [35] reported good fit indices in the Spanish version of the CAPL-2, supporting its validity in assessing physical literacy and predicting physical activity levels. In fact, the model showed better predictive capabilities for children meeting at least 5 days of physical activity, indicating the questionnaire’s strong discriminative power, particularly among children closely adhering to minimum recommended activity levels. These findings confirm the CAPL-2’s utility as a practical screening instrument in identifying physically inactive children and underscore its potential to guide targeted interventions.

Figure 1 visually supports the results, clearly displaying excellent sensitivity and specificity of the CAPL-2 questionnaire for predicting adherence to recommended activity levels, further reinforcing the quantitative evidence provided by Table 5. The curve’s proximity to the top-left corner indicates optimal discriminatory performance, confirming the questionnaire’s appropriateness for practical use in educational and health-related settings in Italy.

In the context of Italy’s recent educational reforms in the PA and sport contexts, including the introduction of specialized physical education teachers in primary schools (Legge 234/2021) [36] the validation of CAPL-2 questionnaire could offer a valuable and culturally adapted instrument to trainers, teachers, educators and policymakers, enabling effective monitoring of PL and targeted interventions from an early age. By facilitating early identification of areas needing support, this instrument contributes to the establishment of active lifestyle trajectories that could persist throughout the lifespan [48,49]. In fact, motor illiteracy complicates motor development by reducing competence and confidence, often worsened by obesity [48]. This creates a barrier that negatively impacts emotional, behavioral, physical, and cognitive domains. Effective physical education strategies that promote autonomy, motivation, and confidence can increase enjoyment and perceived competence, encouraging long-term participation in physical activities [50,51].

The present study’s purpose also aligns with broader European efforts promoting physical literacy, including the Erasmus+ project “Promoting Physical Literacy and Healthy Lifestyles through Digital Materials for University Students (ePhyLi)” (https://www.ephyliproject.eu/), involving Italian institutions. Furthermore, it aligns with global sustainability and health objectives, such as those outlined in the UN 2030 Agenda [52], fostering healthier lifestyles from childhood into adulthood and recognizing sport and physical activity as catalysts for health, education, inclusion, and equality. Our study supports this perspective by providing a validated tool to monitor and promote these outcomes through physical literacy.

Moreover, our study aligns with the Kazan Action Plan [53] that provides a relevant international framework emphasizing inclusive access for all to physical education, physical activity, and sport, with the specific aim to maximize contributions to sustainable development goals (SDGs). Among the Kazan priorities, our study specifically addresses objectives related to improving health and well-being (SDG 3), providing quality education and lifelong learning (SDG 4), fostering inclusive participation (SDG 10), and advancing gender equality (SDG 5). Furthermore, the Kazan Action Plan underscores the importance of using evidence-based tools, such as CAPL-2, for monitoring and evaluating policies and programs designed to enhance physical literacy, physical activity, and sport participation.

## 5. Limitations

While the present study provides a reliable and contextually adapted tool for assessing and promoting PL among Italian children, certain limitations should be considered when interpreting the findings. Although the sample size was sufficient to estimate internal consistency with confidence and is comparable to that used in other CAPL-2 questionnaire adaptations [17,32,33] it was not adequate to perform advanced factorial analyses such as Confirmatory Factor Analysis (CFA). Moreover, the use of a convenience sample from a national summer camp, while providing diversity in socio-cultural backgrounds, may limit the generalizability of the results to the broader pediatric population. In addition, the cross-sectional design did not allow for the assessment of long-term stability or predictive validity of the CAPL-2 questionnaire. Future research should involve larger, randomized, and demographically diverse samples to enhance external validity, and adopt longitudinal or intervention-based designs to examine how early PL levels influence sustained PA and sport participation across the lifespan.

## 6. Conclusions

To our knowledge, this is the first study adapting and validating the CAPL-2 questionnaire for Italian children aged 8 to 12 years, providing a reliable and contextually appropriate instrument to assess PL. It represents a significant advancement in aligning assessment tools with the holistic and inclusive conceptualization of PL [15], facilitating culturally tailored interventions aimed at lifelong engagement in physical activity.

The strong internal consistency observed in the Motivation and Confidence domain underscores the relevance of affective components in fostering sustained physical activity behaviors, supporting multicomponent interventions addressing these motivational dimensions [32,43]. Conversely, the lower reliability observed in the Knowledge and Understanding domain, likely due to limited variability among participants with high baseline knowledge, highlights the necessity for further refinement to ensure comprehensive assessment across all PL domains. These results suggest that future research should explore revised formulations or expanded item pools to enhance the psychometric performance of this domain.

## Figures and Tables

**Table 1:** Sample size and demographic characteristics.

Variable	Mean	Standard Deviation	Min	Max
Weight (kg)	34.64	9.38	19.9	64.4
Height (cm)	137.86	10.27	119.4	159.2
BMI (kg/cm^2^)	17.93	3.08	13.21	26.98
Active days (days/week)	5.69	1.36	1	7

**Table 2:** Reliability, test-retest and systematic differences in motivation and confidence questions from CAPL-2 battery assessment.

Motivation and Confidence	Mean (SD)	Item-Total Correlation (Test)	Item-Total Correlation (Retest)	Average Inter-Item Correlation	Cronbach’s α
Item 1	2.19 (0.56)	0.97	0.92	0.79	0.88
Item 2	2.12 (0.63)	0.91	0.80	0.95	0.97
Item 3	2.15 (0.56)	0.96	0.92	0.79	0.89
Predilection	6.45 (1.67)	N/A	N/A	0.84	0.94
Item 1	1.96 (0.63)	0.91	0.79	0.87	0.93
Item 2	1.75 (0.64)	0.94	0.85	0.78	0.88
Item 3	1.87 (0.63)	0.95	0.88	0.75	0.85
Adequacy	5.59 (1.77)	N/A	N/A	0.80	0.92
Item 1	2.20 (0.43)	0.92	0.83	0.93	0.96
Item 2	2.04 (0.44)	0.97	0.93	0.93	0.96
Item 3	1.99 (0.42)	0.96	0.91	0.83	0.91
Intrinsic motivation	6.23 (1.22)	N/A	N/A	0.86	0.95
Item 1	1.89 (0.49)	0.97	0.93	0.82	0.90
Item 2	1.57 (0.52)	0.93	0.84	0.94	0.97
Item 3	1.83 (0.50)	0.96	0.92	0.83	0.91
Physical activity competence	5.30 (1.45)	N/A	N/A	0.86	0.95
Total domain score	23.51 (5.65)	N/A	N/A	0.74	0.97

Abbreviations: SD, standard deviation; N/A, not applicable. Item-total correlation refers to the magnitude of association between each item with its domain. Cronbach’s a refers to the value when the item is removed.

**Table 3:** Reliability, test-retest and systematic differences in knowledge and understanding questions from CAPL-2 battery assessment.

Knowledge and Understanding	Mean (SD)	Item-Total Correlation (Test)	Item-Total Correlation (Retest)	Average Inter-Item Correlation	Cronbach’s α
Physical activity (PA) guidelines	0.97 (0.19)	0.61	0.10	0.11	0.20
Cardiorespiratory fitness definition	1 (0)	N/A	N/A	N/A	N/A
Muscular endurance definition	1 (0)	N/A	N/A	N/A	N/A
Improve sport skills	0.53 (0.50)	0.70	0.23	0.21	0.34
PA comprehension	5.80 (0.60)	0.56	0.03	0.21	0.34
Total domain score	9.30 (0.86)	N/A	N/A	0.08	0.22

Abbreviations: SD, standard deviation; N/A, not applicable. Item-total correlation refers to the magnitude of association between each item with its domain. Cronbach’s a refers to the value when the item is removed.

**Table 4:** Correlations within the Motivation and Confidence domains.

	1	2	3	4
1. Predilection	-			
2. Adequacy	0.77 *	-		
3. Intrinsic Motivation	0.83 *	0.74 *	-	
4. PA competence	0.77 *	0.88 *	0.80 *	-
Motivation and Confidence	0.92 *	0.93 *	0.90 *	0.94 *

* *p* < 0.05.

**Table 5:** Results of ROC curve analysis for CAPL-2 Questionnaire total score based on children’s physical activity.

MVPA Cut Point	Coefficient	St.Err.	z	*p*	[95% CI]		AUC (95% CI)	*p*
Active for at least 6 days	0.33	0.65	7.07	<0.001	0.20	0.46	0.89	<0.001
Active for at least 5 days	0.42	0.09	4.69	<0.001	0.24	0.59	0.95	<0.001

## Data Availability

The data are available from the corresponding author on reasonable request.

## Supplementary Materials

The following supporting information can be downloaded at: https://www.mdpi.com/article/10.3390/children12101290/s1. Figure S1: Graphical representation of the process of translation and cultural adaptation of the Italian version of the Canadian Physical Literacy Assessment-2 questionnaire; Supplementary Material S1: Italian version of the CAPL-2 questionnaire.

## References

[1] Boer M., van den Eijnden R.J.J.M., Boniel-Nissim M., Wong S.L., Inchley J.C., Badura P., Craig W.M., Gobina I., Kleszczewska D., Klanšček H.J. (2020). Adolescents’ Intense and Problematic Social Media Use and Their Well-Being in 29 Countries. J. Adolesc. Health.

[2] Galeotti T., Canale N., Charrier L., Bacigalupo I., Lazzeri G., Vieno A. (2024). La Sorveglianza HBSC-Italia 2022 Health Behaviour in School-Aged Children: I Comportamenti Di Dipendenza.

[3] Kern M.R., Duinhof E.L., Walsh S.D., Cosma A., Moreno-Maldonado C., Molcho M., Currie C., Stevens G.W.J.M. (2020). Intersectionality and Adolescent Mental Well-Being: A Cross-Nationally Comparative Analysis of the Interplay Between Immigration Background, Socioeconomic Status and Gender. J. Adolesc. Health.

[4] Digennaro S., Iannaccone A. (2025). Imagining Another Self: The Use of Social Media Among Preadolescents and Its Body-Related Consequences. An Exploratory Study. Sage Open.

[5] Digennaro S., Iannaccone A. (2023). Check Your Likes but Move Your Body! How the Use of Social Media Is Influencing Pre-Teens Body and the Role of Active Lifestyles. Sustainability.

[6] Piccerillo L., Tescione A., Iannaccone A., Digennaro S. (2025). Alpha Generation’s Social Media Use: Sociocultural Influences and Emotional Intelligence. Int. J. Adolesc. Youth.

[7] Guthold R., Stevens G.A., Riley L.M., Bull F.C. (2020). Global Trends in Insufficient Physical Activity among Adolescents: A Pooled Analysis of 298 Population-Based Surveys with 1·6 Million Participants. Lancet Child. Adolesc. Health.

[8] Wilhite K., Booker B., Huang B.-H., Antczak D., Corbett L., Parker P., Noetel M., Rissel C., Lonsdale C., del Pozo Cruz B. (2023). Combinations of Physical Activity, Sedentary Behavior, and Sleep Duration and Their Associations With Physical, Psychological, and Educational Outcomes in Children and Adolescents: A Systematic Review. Am. J. Epidemiol..

[9] Bull F.C., Al-Ansari S.S., Biddle S., Borodulin K., Buman M.P., Cardon G., Carty C., Chaput J.-P., Chastin S., Chou R. (2020). World Health Organization 2020 Guidelines on Physical Activity and Sedentary Behaviour. Br. J. Sports Med..

[10] Carl J., Barratt J., Arbour-Nicitopoulos K.P., Barnett L.M., Dudley D.A., Holler P., Keegan R., Kwan M., Scurati R., Sum R.K.-W. (2023). Development, Explanation, and Presentation of the Physical Literacy Interventions Reporting Template (PLIRT). Int. J. Behav. Nutr. Phys. Act..

[11] Dlugonski D., Gadd N., McKay C., Kleis R.R., Hoch J.M. (2022). Physical Literacy and Physical Activity Across the Life Span: A Systematic Review. Transl. J. Am. Coll. Sports Med..

[12] Agosti V., Sirico M. (2020). Motor Imagery as a Tool for Motor Learning and Improving Sports Performance: A Mini Review on the State of the Art. Sport Sci..

[13] Neil-Sztramko S.E., Caldwell H., Dobbins M. (2009). School-Based Physical Activity Programs for Promoting Physical Activity and Fitness in Children and Adolescents Aged 6 to 18. Cochrane Database Syst. Rev..

[14] Farias C., Wallhead T., Mesquita I. (2020). “The Project Changed My Life”: Sport Education’s Transformative Potential on Student Physical Literacy. Res. Q. Exerc. Sport.

[15] Whitehead M. (2019). Definition of Physical Literacy: Development and Issues. Physical Literacy Across the World.

[16] Whitehead M. (2010). Physical Literacy: Throughout the Lifecourse.

[17] Pastor-Cisneros R., Carlos-Vivas J., Adsuar J.C., Barrios-Fernández S., Rojo-Ramos J., Vega-Muñoz A., Contreras-Barraza N., Mendoza-Muñoz M. (2022). Spanish Translation and Cultural Adaptation of the Canadian Assessment of Physical Literacy-2 (CAPL-2) Questionnaires. Int. J. Environ. Res. Public Health.

[18] Rudd J.R., Pesce C., Strafford B.W., Davids K. (2020). Physical Literacy—A Journey of Individual Enrichment: An Ecological Dynamics Rationale for Enhancing Performance and Physical Activity in All. Front. Psychol..

[19] Carl J., Jaunig J., Schnith L., Mayer J., O’Connor J., Young L. (2024). Mapping the ‘Lifelong Journey’ of Physical Literacy: A Biographical Assessment Method for the Physical Activity and Health Context. Sport Educ. Soc..

[20] Hadier S.G., Liu Y., Long L., Hamdani S.M.Z.H., Khurram H., Hamdani S.D., Danish S.S., Fatima S.U. (2024). Assessment of Physical Literacy in 8- to 12-Year-Old Pakistani School Children: Reliability and Cross-Validation of the Canadian Assessment of Physical Literacy-2 (CAPL-2) in South Punjab, Pakistan. BMC Public Health.

[21] Dania A., Kaioglou V., Venetsanou F. (2020). Validation of the Canadian Assessment of Physical Literacy for Greek Children: Understanding Assessment in Response to Culture and Pedagogy. Eur. Phys. Educ. Rev..

[22] Barnett L.M., Jerebine A., Keegan R., Watson-Mackie K., Arundell L., Ridgers N.D., Salmon J., Dudley D. (2023). Validity, Reliability, and Feasibility of Physical Literacy Assessments Designed for School Children: A Systematic Review. Sports Med..

[23] Boldovskaia A., Dias N.M.G., Silva M.N., Carraça E.V. (2023). Physical Literacy Assessment in Adults: A Systematic Review. PLoS ONE.

[24] Carl J., Barratt J., Wanner P., Töpfer C., Cairney J., Pfeifer K. (2022). The Effectiveness of Physical Literacy Interventions: A Systematic Review with Meta-Analysis. Sports Med..

[25] Pascali G., Colella D. (2025). Physical Literacy Assessment. A Literature Review. Eur. J. Phys. Educ. Sport Sci..

[26] Edwards L.C., Bryant A.S., Keegan R.J., Morgan K., Jones A.M. (2017). Definitions, Foundations and Associations of Physical Literacy: A Systematic Review. Sports Med..

[27] World Health Organization (2018). Global Action Plan on Physical Activity 2018–2030: More Active People for a Healthier World.

[28] Carl J., Mazzoli E., Mouton A., Sum R.K.-W., Singh A., Niederberger M., Martins J., Kriellaars D., Green N., Elsborg P. (2024). Development of a Global Physical Literacy (GloPL) Action Framework: Study Protocol for a Consensus Process. PLoS ONE.

[29] Longmuir P.E., Gunnell K.E., Barnes J.D., Belanger K., Leduc G., Woodruff S.J., Tremblay M.S. (2018). Canadian Assessment of Physical Literacy Second Edition: A Streamlined Assessment of the Capacity for Physical Activity among Children 8 to 12 Years of Age. BMC Public Health.

[30] Li M.H., Sum R.K.W., Tremblay M., Sit C.H.P., Ha A.S.C., Wong S.H.S. (2020). Cross-Validation of the Canadian Assessment of Physical Literacy Second Edition (CAPL-2): The Case of a Chinese Population. J. Sports Sci..

[31] Vuletic P.R., Kesic M.G., Gilic B., Pehar M., Uzicanin E., Idrizovic K., Sekulic D. (2023). Evaluation of Physical Literacy in 9- to 11-Year-Old Children: Reliability and Validity of Two Measurement Tools in Three Southeastern European Countries. Children.

[32] Elsborg P., Melby P.S., Kurtzhals M., Tremblay M.S., Nielsen G., Bentsen P. (2021). Translation and Validation of the Canadian Assessment of Physical Literacy-2 in a Danish Sample. BMC Public Health.

[33] Knisel E., Bremer M., Nałęcz H., Wascher L., Laudańska-Krzemińska I. (2024). Validation of The Canadian Assessment of Physical Literacy—CAPL-2 Questionnaire for German and Polish School Children. Phys. Cult. Sport.

[34] Mendoza-Muñoz M., Carlos-Vivas J., Castillo-Paredes A., Parraca J.A., Raimundo A., Alegrete J., Pastor-Cisneros R., Gomez-Galan R. (2023). Portuguese Translation and Validation of the Questionnaires from the Canadian Physical Literacy Assessment-2: A Pilot Study. Front. Psychol..

[35] Mendoza Muñoz M., López-Gil J.F., Pastor-Cisneros R., Castillo Paredes A., Urbano Mairena J., Tremblay M., Carlos Vivas J. (2024). Cross-Validation of the Canadian Assessment of Physical Literacy Second Edition (CAPL-2) for Spanish Children. BMJ Open Sport Exerc. Med..

[36] Ministero Dell’Istruzione Dipartimento per Il Sistema Educativo Di Istruzione e Formazione. Gazzetta Ufficiale Della Repubblica. Legge 30 Dicembre 2021, n. 234, Bilancio Di Previsione Dello Stato per l’anno Finanziario 2022 e Bilancio Pluriennale per Il Triennio 2022-2024, Art. 1, Co. 332, in G.U., Serie Generale, n. 310 Del 31.12.2021, S.O. n. 49. https://www.gazzettaufficiale.it/eli/id/2021/12/31/21G00256/s.

[37] Longmuir P.E., Woodruff S.J., Boyer C., Lloyd M., Tremblay M.S. (2018). Physical Literacy Knowledge Questionnaire: Feasibility, Validity, and Reliability for Canadian Children Aged 8 to 12 Years. BMC Public Health.

[38] Walde P., Völlm B.A. (2023). The TRAPD Approach as a Method for Questionnaire Translation. Front. Psychiatry.

[39] Nunnally J.C. (1978). Psychometric Theory (2nd...—Google Scholar). https://scholar.google.com/scholar?hl=it&as_sdt=0%2C5&q=Nunnally%2C+J.+C.+%281978%29.+Psychometric+theory+%282nd+ed.%29.+McGraw-Hill.&btnG=.

[40] George D. (2011). SPSS for Windows Step by Step: A Simple Study Guide and Reference, 17.0 Update, 10/e..

[41] Landis J.R., Koch G.G. (1977). The Measurement of Observer Agreement for Categorical Data. Biometrics.

[42] Polo T.C.F., Miot H.A. (2020). Use of ROC Curves in Clinical and Experimental Studies. J. Vasc. Bras..

[43] Gunnell K.E., Longmuir P.E., Woodruff S.J., Barnes J.D., Belanger K., Tremblay M.S. (2018). Revising the Motivation and Confidence Domain of the Canadian Assessment of Physical Literacy. BMC Public Health.

[44] Cudicio A., Agosti V. (2024). Beyond Belief: Exploring the Alignment of Self-Efficacy, Self-Prediction, Self-Perception, and Actual Performance Measurement in a Squat Jump Performance—A Pilot Study. J. Funct. Morphol. Kinesiol..

[45] Kyriazos T.A. (2018). Applied Psychometrics: Sample Size and Sample Power Considerations in Factor Analysis (EFA, CFA) and SEM in General. Psychology.

[46] Worthington R.L., Whittaker T.A. (2006). Scale Development Research. Couns. Psychol..

[47] Terwee C.B., Bot S.D.M., de Boer M.R., van der Windt D.A.W.M., Knol D.L., Dekker J., Bouter L.M., de Vet H.C.W. (2007). Quality Criteria Were Proposed for Measurement Properties of Health Status Questionnaires. J. Clin. Epidemiol..

[48] Colella D. (2018). Physical Literacy e Stili d’insegnamento. Ri-Orientare l’educazione Fisica a Scuola Physical Literacy and Teaching Styles. Re-Orienting Physical Education at School. Form. Insegn..

[49] Monacis D., Sannicandro I., Colella D. (2025). Exploring Self-Reported Physical Activity Levels and Physical Fitness in Italian Children: A Mediation and Moderation Analysis. Children.

[50] Trecroci A., Invernizzi P.L., Monacis D., Colella D. (2021). Physical Illiteracy and Obesity Barrier: How Physical Education Can Overpass Potential Adverse Effects? A Narrative Review. Sustainability.

[51] Faella P., Digennaro S., Iannaccone A. (2025). Educational Practices in Motion: A Scoping Review of Embodied Learning Approaches in School. Front. Educ..

[52] United Nation Agenda Sustainable Development 2030. https://www.un.org/sustainabledevelopment/.

[53] UNESCO UNESCO Kazan Action Plan. Proceedings of the International Conference of Ministers and Senior Officials Responsible for Physical Education and Sport, 6th.

