# Supplementary material for: Validation of the Italian Translation and Cultural Adaptation of the Canadian Assessment of Physical Literacy-2 (CAPL-2) Questionnaire for Children
Source: Children (Basel). 2025 Sep 24;12(10):1290. doi: 10.3390/children12101290 (PMC12563355; doi:10.3390/children12101290)
Supplement: Supplementary file 1 [file children-12-01290-s001.zip › children-3831794-CAPL-2-questionnaire_ITA_def-04.08.2025.pdf]

Questionario CAPL–2
Valutazione Canadese della Physical Literacy- 2
(Versione Italiana)

Cosa ne pensi dell'Attività Fisica?
Quando parliamo di attività fisica, facciamo riferimento a quando ti muovi, giochi o fai esercizio fisico. L'attività fisica è tutto quello che fai e che ti fa battere il cuore più veloce o ti fa restare un po' senza fiato.

Perché ti facciamo queste domande?
Ci piacerebbe sapere cosa pensano ragazzi e ragazze come te dell'attività fisica, dello sport e dell'esercizio fisico in generale.

Per favore, ricorda:

- Non ci sono risposte giuste o sbagliate! Vogliamo solo sapere cosa ne pensi.
- Se non conosci la risposta, scegli quella che ti sembra giusta.
- Non c'è un limite di tempo per rispondere, prendi il tempo di cui hai bisogno.

Cosa mi rappresenta di più?

Per ogni domanda, leggi le due frasi e cerchia **QUELLA CHE PENSI TI RAPPRESENTI DI PIU'.**

Prova con questo **ESEMPIO DI DOMANDA #1**

**Alcuni bambini hanno un solo naso sul viso**	**MA**	**Altri bambini ne hanno tre**

Non dovrebbe essere troppo difficile scegliere!

Una volta che hai cerchiato la frase che più <u>ti rappresenta</u>, ti verrà chiesto di indicare se <u>per te</u> è **MOLTO VERO** o **ABBASTANZA VERO**.

Per esercitarti, di seguito ti proponiamo un'altra domanda d'esempio. Ricorda, per rispondere alle domande è necessario fare due cose:

1) **<u>Prima cerchia la frase che più ti rappresenta.</u>**

2) **<u>Poi, segna con una X se per te la frase è MOLTO VERA o solo ABBASTANZA VERA.</u>**

NON ESISTONO RISPOSTE GIUSTE O SBAGLIATE, CERCHIA SOLO QUALE FRASE PENSI CHE **TI RAPPRESENTI DI PIU'!**

Prova con questo **esempio di domanda #2**

**Ad alcuni/e ragazzi/e piace giocare con i dispositivi digitali**		**MA**	**Ad altri/e ragazzi/e non piace giocare con i dispositivi digitali**	
**□ MOLTO VERO per me**	**□ ABBASTANZA VERO per me**		**□ MOLTO VERO per me**	**□ ABBASTANZA VERO per me**

Ora sei pronto/a per iniziare compilare il questionario. **Ricorda, in ogni riquadro devi cerchiare la frase che ti rappresenta di più e poi mettere una X sulla casella "molto vero" oppure "abbastanza vero".** Prendi tutto il tempo che ti serve e procedi con attenzione. Se hai qualche dubbio, chiedi pure! Se pensi di essere pronto/a, puoi cominciare!

CONTROLLA BENE DI AVER RISPOSTO SU OGNI PAGINA!

Cosa mi rappresenta di più?

**Ad alcuni/e ragazzi/e non piace giocare ai giochi attivi**		**MA**	**Ad altri/e ragazzi/e piace molto giocare ai giochi attivi**	
**□ MOLTO VERO per me**	**□ ABBASTANZA VERO per me**		**□ MOLTO VERO per me**	**□ ABBASTANZA VERO per me**

**Alcuni/e ragazzi/e sono bravi/e nei giochi attivi**		**MA**	**Altri/e ragazzi/e trovano difficile giocare ai giochi attivi**	
**□ MOLTO VERO per me**	**□ ABBASTANZA VERO per me**		**□ MOLTO VERO per me**	**□ ABBASTANZA VERO per me**

**Alcuni/e ragazzi/e non si divertono molto a praticare sport**		**MA**	**Altri/e ragazzi/e si divertono molto a praticare sport**	
**□ MOLTO VERO per me**	**□ ABBASTANZA VERO per me**		**□ MOLTO VERO per me**	**□ ABBASTANZA VERO per me**

**Alcuni/e ragazzi/e sono bravi/e nella maggior parte degli sport**		**MA**	**Altri/e ragazzi/e pensano di non essere bravi bravi/e negli sport**	
**□ MOLTO VERO per me**	**□ ABBASTANZA VERO per me**		**□ MOLTO VERO per me**	**□ ABBASTANZA VERO per me**

**Ad alcuni/e ragazzi/e non piace fare sport**		**MA**	**Ad altri/e ragazzi/e piace molto fare sport**	
**□ MOLTO VERO per me**	**□ ABBASTANZA VERO per me**		**□ MOLTO VERO per me**	**□ ABBASTANZA VERO per me**

**Alcuni/e ragazzi/e imparano facilmente a giocare ai giochi attivi**		**MA**	**Altri/e ragazzi/e trovano difficile imparare a giocare ai giochi attivi**	
**□ MOLTO VERO per me**	**□ ABBASTANZA VERO per me**		**□ MOLTO VERO per me**	**□ ABBASTANZA VERO per me**

Grazie per averci indicato chi ti rappresenta di più!

Abbiamo ancora qualche domanda sull'attività fisica. Procedi con la pagina successiva.

Perché sei attivo/a?

I ragazzi e le ragazze possono essere attivi/e partecipando a diverse attività:

- Facendo esercizio fisico (come camminare, allenarsi o partecipare a lezioni di ginnastica)
- Giocando all'aperto o praticando attività che richiedono impegno fisico (come giocare al parco)
- Praticando sport (come calcio, tennis, pallavolo, danza o nuoto)

Di seguito troverai alcuni motivi per cui potresti essere attivo/a.

Per favore, leggi ogni frase e per ognuna dicci quanto è vera per te.

**Sono attivo/a perché…**					
	Non è vero per me	Non è del tutto vero per me	A volte è vero per me	Spesso è vero per me	È molto vero per me
essere attivo/a è divertente	☐	☐	☐	☐	☐
ho piacere ad essere attivo/a	☐	☐	☐	☐	☐
mi piace essere attivo/a	☐	☐	☐	☐	☐

Che cosa provi quando sei attivo/a?

Nella sezione successiva troverai alcune frasi che descrivono come si sentono i ragazzi e le ragazze QUANDO SONO ATTIVI e QUANDO FANNO ATTIVITA' CHE RICHIEDONO IMPEGNO FISICO (come giocare ai giochi attivi, giocare all'aperto o praticare sport). Per favore, leggi ogni frase e per ognuna indica quanto ti rappresenta.

	Non mi rappresenta per niente	Non mi rappresenta del tutto	Mi rappresenta a volte	Mi rappresenta abbastanza	Mi rappresenta molto
Quando capita di giocare ai giochi attivi, penso di essere abbastanza bravo/a.	☐	☐	☐	☐	☐
Rispetto agli altri/e, penso di essere bravo/a nelle attività.	☐	☐	☐	☐	☐
Quando sono attivo/a, ho delle buone abilità.	☐	☐	☐	☐	☐

Cosa sai sull'attività fisica?

Per favore, per ogni domanda cerchia una sola risposta

1. Quanti minuti al giorno tu e gli altri ragazzi/e dovreste dedicare ad attività fisiche che facciano accelerare il battito del cuore ed il respiro, come ad esempio camminare velocemente o correre? Conta anche il tempo in cui dovresti essere attivo a scuola, a casa, o nel tuo quartiere.

a) 20 minuti

b) 30 minuti

c) 60 minuti o 1 ora

d) 120 minuti o 2 ore

2. Esistono diverse tipologie di efficienza fisica. Una di queste è nota come resistenza cardiovascolare, efficienza aerobica, o fitness cardiorespiratorio. Per fitness cardiorespiratorio si intende:

a) La capacità dei muscoli di spingere, tirare o allungarsi
b) La capacità del cuore di pompare il sangue e dei polmoni di fornire ossigeno
c) Avere un peso adeguato alla propria altezza
d) La nostra capacità di praticare gli sport che ci piacciono

3. Per forza muscolare o resistenza muscolare si intende:

a) La capacità dei muscoli di spingere, tirare o allungarsi
b) La capacità del cuore di pompare il sangue e dei polmoni di fornire ossigeno
c) Avere un peso adeguato alla propria altezza
d) La nostra capacità di praticare gli sport che ci piacciono

4. Se tu volessi migliorare IN UNA ABILITÀ SPORTIVA (come calciare o afferrare una palla), quale sarebbe la cosa migliore da fare?

a) Leggere un libro sul calciare e afferrare una palla

b) Aspettare di diventare più grande

c) Fare più esercizio fisico o essere più attivo/a

d) Guardare un video, seguire una lezione, o farsi aiutare da un allenatore per imparare a calciare e afferrare una palla

5. In questa storia su Sara mancano alcune parole. Completa le frasi utilizzando le parole che trovi nella casella sottostante. Per riempire uno spazio vuoto, ogni parola può essere usata una sola volta. Ci sono più parole che spazi vuoti, quindi, alcune parole non saranno utilizzate.

Divertente		Allungamento (stretching)	Resistenza
Battito	Respiro	Flessibilità	Buona
Forza	Male	Sport	

Sara cerca di essere attiva ogni giorno. Correre ogni giorno fa bene al suo cuore e ai suoi polmoni. Sara ritiene che l'attività fisica sia ___________ed anche ____________ per lei. Durante i suoi allenamenti con la squadra, corre di più per migliorare la sua ___________. Durante gli allenamenti con la squadra fa anche esercizi come piegamenti e addominali per aumentare la sua ___________.
Durante il defaticamento, Sara fa esercizi di _______ per migliorare la sua flessibilità e abbassare la sua frequenza cardiaca. Dopo il suo allenamento, Sara monitora la sua frequenza cardiaca, anche nota come___.

6. Durante la scorsa settimana (negli ultimi 7 giorni), in quanti giorni sei stato/a fisicamente attivo/a per un totale di almeno 60 minuti al giorno? Conta tutto il tempo che hai dedicato ad attività che hanno fatto aumentare la tua frequenza cardiaca o che ti hanno fatto respirare affannosamente.

Sono stato/a attivo/a 0 1 2 3 4 5 6 7 giorni

Parlaci di te!

<u>Per favore cerchia un solo numero, o parola, o scelta per ogni domanda</u>

A scuola quale classe frequenti?

Se in questo momento non vai a scuola, cerchia la classe che frequenterai il prossimo anno scolastico.

- Terza classe scuola primaria (Terza elementare)
- Quarta classe scuola primaria (Quarta elementare)
- Quinta classe scuola primaria (Quinta elementare)
- Prima classe scuola secondaria di primo grado (Prima media)
- Seconda classe scuola secondaria di primo grado (Seconda media)
- Terza classe scuola secondaria di primo grado (Terza media)

Sei:

Ragazzo Ragazza

In quale mese sei nato?

Gen Feb Mar Apr Mag Giu Lug Ago Set Ott Nov Dic

Quanti anni hai?

7 8 9 10 11 12 13

Grazie per aver partecipato!

☺
