# Supplementary material for: Validation of the Italian Translation and Cultural Adaptation of the Canadian Assessment of Physical Literacy-2 (CAPL-2) Questionnaire for Children
Source: Children (Basel). 2025 Sep 24;12(10):1290. doi: 10.3390/children12101290 (PMC12563355; doi:10.3390/children12101290)
Supplement: Supplementary file 1 [file children-12-01290-s001.zip › children-3831794-supplementary(2).docx]

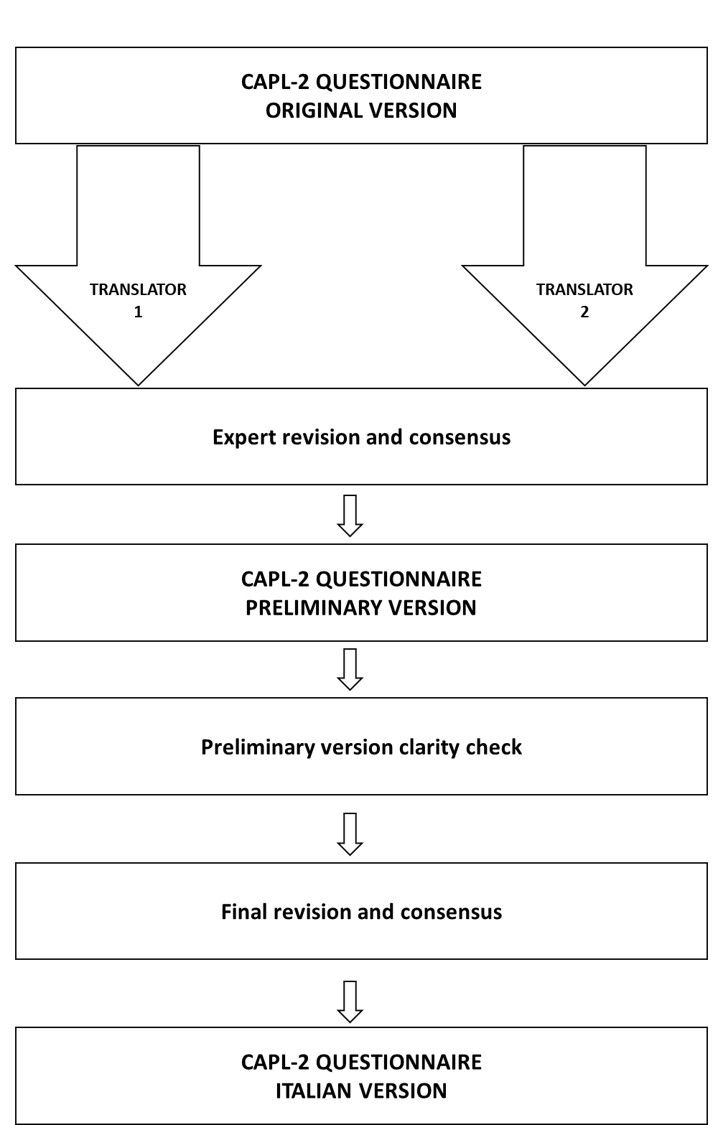


**Figure S1.** Graphical representation of the process of translation and cultural adaptation of the Italian version of the Canadian Physical Literacy Assessment-2 questionnaire.
